# Many rivers to cross: the journey of zinc from soil to seed

**DOI:** 10.3389/fpls.2014.00030

**Published:** 2014-02-12

**Authors:** Lene I. Olsen, Michael G. Palmgren

**Affiliations:** ^1^Centre for Membrane Pumps in Cells and Disease-PUMPKIN, Danish National Research FoundationFrederiksberg, Denmark; ^2^Copenhagen Plant Science Centre, Department of Plant and Environmental Sciences, University of CopenhagenFrederiksberg, Denmark

**Keywords:** plants, seeds, apoplastic barrier, zinc, nicotianamine

## Abstract

An important goal of micronutrient biofortification is to enhance the amount of bioavailable zinc in the edible seed of cereals and more specifically in the endosperm. The picture is starting to emerge for how zinc is translocated from the soil through the mother plant to the developing seed. On this journey, zinc is transported from symplast to symplast via multiple apoplastic spaces. During each step, zinc is imported into a symplast before it is exported again. Cellular import and export of zinc requires passage through biological membranes, which makes membrane-bound transporters of zinc especially interesting as potential transport bottlenecks. Inside the cell, zinc can be imported into or exported out of organelles by other transporters. The function of several membrane proteins involved in the transport of zinc across the tonoplast, chloroplast or plasma membranes are currently known. These include members of the ZIP (ZRT-IRT-like Protein), and MTP (Metal Tolerance Protein) and heavy metal ATPase (HMA) families. An important player in the transport process is the ligand nicotianamine that binds zinc to increase its solubility in living cells and in this way buffers the intracellular zinc concentration.

## INTRODUCTION

It is estimated that one-third of the world’s population suffers from zinc deficiency with serious consequences (Sandstead, 1991; Welch and Graham, 1999, 2004; World Health Organization, 2003). Zinc deficiency is especially pronounced in areas where people are relying on a plant-based diet, as the edible parts of plants have a low content of bioavailable micronutrients. In cereal grains, high concentrations of zinc are found in the embryo and the aleurone layer of the endosperm (Lombi et al., 2011; Lu et al., 2013a). The localization of zinc is of concern since the embryo and aleurone layer are removed during the polishing of the grains. It is therefore of interest to produce grains with a higher concentration of zinc in the edible endosperm.

Plants, like humans and any other organism, rely on a sufficient zinc supply to drive cellular functions. More than a thousand different proteins have been found to be associated with zinc for functionality in *Arabidopsis thaliana* (Krämer et al., 2007). Zinc serves catalytic, regulatory, and structural roles for a great number of proteins and enzymes with one of the biggest classes of zinc-requiring proteins being the zinc-finger transcription factors (Broadley et al., 2007). Enzymes involved in the synthesis and maintenance of DNA and RNA also requires zinc and the copper/zinc superoxide dismutase in the chloroplast stroma is another example (Hansch and Mendel, 2009). However, in excess amounts zinc is able to replace other metals or bind to undesired proteins and enzymes resulting in their inactivation. Thus, zinc is essential for cellular functions but is toxic at high concentrations. Therefore a tightly controlled homeostatic network consisting of import, trafficking, sequestration and export is needed for the plant to survive (Clemens, 2001; Clemens et al., 2002; Hall, 2002).

**FIGURE 1:**
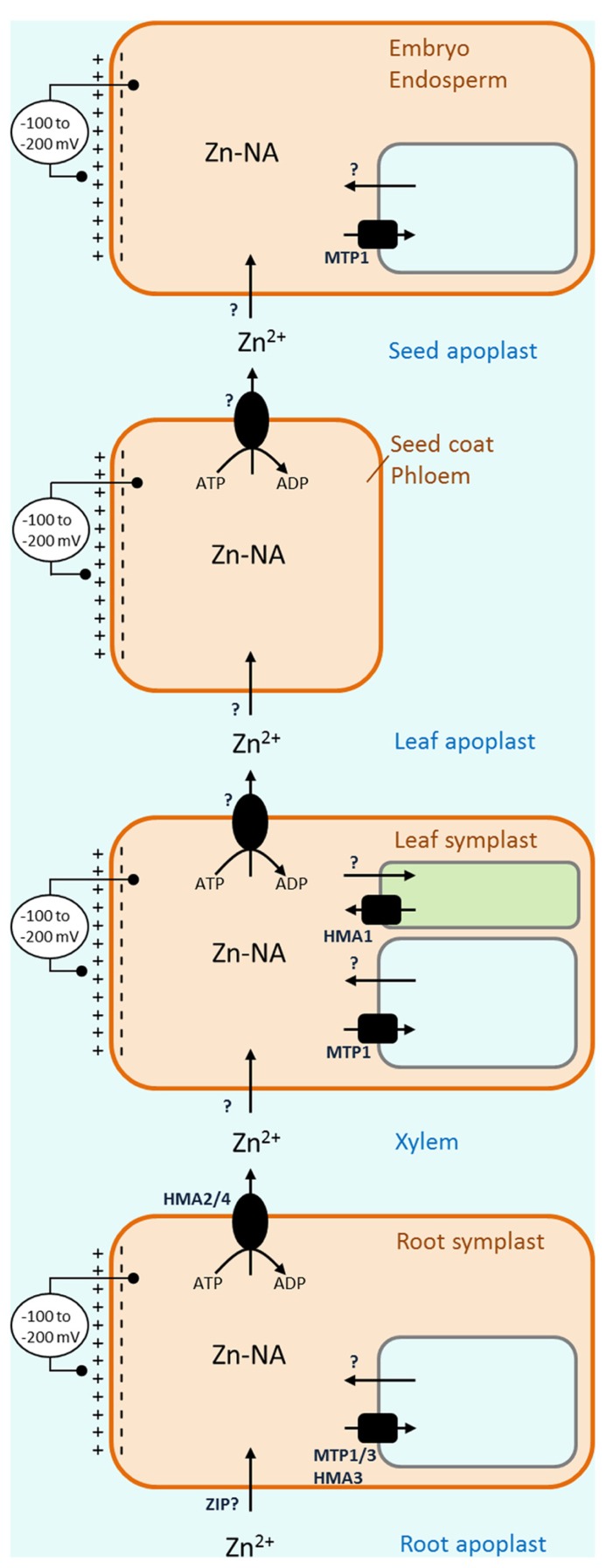
** Overview of transport barriers leading to loading of zinc into seeds.** Following uptake of zinc into the root symplast, at least three apoplastic barriers have to be crossed on the way to the seed. Substantial membrane potentials cross the membranes of the plasma membrane, vacuole and chloroplast, transport of zinc into the cytoplasm, out of the vacuole or into the chloroplast is energetically favorable, requiring passive transporters only (arrows). On the other hand, transport out of the cytosol, into the vacuole or out of the chloroplast requires active transporters or secondary active transporters (round and square symbols, respectively).

Once zinc is taken up into roots it enters a symplast, a living interconnected networks of cells. However, the long way for zinc to the developing seed requires multiple steps where zinc has to move from symplast to symplast. During this process it first has to leave the symplast and enter dead space outside cells, the apoplast, before it can be taken up in a new symplast (**Figure 1**). This transport into and out of the apoplast seems to be the major bottleneck in the process of nutrient translocation within the plant (Palmgren et al., 2008).

A major feature of the plasma membrane of living cells is the presence of a membrane potential, negative on the inside. This membrane potential is maintained by the plasma membrane H^+^-ATPase (Sondergaard et al., 2004), and is a main driving force behind passive cellular uptake of positively charged cations. In *Arabidopsis*, membrane potentials of root cells vary typically between -100 and -115 mV (Bose et al., 2010; Kenderesová et al., 2012) and have been reported as high as above -200 mV (Hirsch et al., 1998). According to Nernst equation (and assuming standard conditions) a membrane potential in this range can sustain a hundred-fold concentration difference of a monovalent cation between the outside and the inside of a cell, and a remarkable ten thousand-fold concentration difference of a divalent cation. With 30 μM of Zn^2^^+^ in the growth medium (as in Murashige and Skoog medium; Murashige and Skoog, 1962) and an inside membrane potential of -115 mV, the cells would in theory by passive uptake be able to establish a cytoplasmic concentration of around 300 mM Zn^2^^+^, which would be far above the cellular requirement and in fact highly toxic. It is therefore evident that uptake of zinc is not an energetic problem and that this process does not require active transport. What is an advantage for uptake of Zn^2^^+^, however, becomes a major disadvantage when Zn^2^^+^ has to be exported from cells. Now the membrane potential, positive on the outside, is a strong opposing factor for outward transport of a divalent cation, and a primary active transport system is required. The vacuole has an inside positive membrane potential requiring active transport systems for import and making export from the vacuole energetically favorable.

## ZINC UPTAKE INTO ROOTS

The first symplastic domain zinc has to enter is that of the root cortex. Uptake into this symplast is likely to be carried out by transport proteins in the plasma membrane of root epidermal or cortical cells. In *Arabidopsis*, IRT1 is a broad substrate range metal ion transporter localized to the plasma membrane that can transport iron (Fe^2^^+^), zinc (Zn^2^^+^), manganese (Mn^2^^+^), cobalt (Co^2^^+^) and cadmium (Cd^2^^+^) (Korshunova et al., 1999) and is subject to intricate metal-dependent post-translational regulation (Shin et al., 2013). This transporter is expressed in the plasma membrane of root epidermal cells (Vert et al., 2002) and is therefore likely to be involved in zinc uptake. Excess Zn causes reduced Fe uptake and excess Fe alleviates Zn toxicity under these conditions, which supports the view that Zn and Fe compete for the same uptake system(s) (Fukao et al., 2011; Shanmugam et al., 2011, 2013). A closely related broad affinity metal transporter, IRT2, is also expressed in epidermal cells (Vert et al., 2001) but in internal membranes (Vert et al., 2009), which excludes a role in direct uptake. Other members of the ZIP (*Z*RT-*I*RT-like *P*rotein) family of metal transporters, to which IRT1 and IRT2 belong, have been proposed to import zinc into roots (Grotz et al., 1998; Lin et al., 2009) but this assumption has recently been questioned as functional studies with plants mutated in the major root *ZIP* genes, At*ZIP1,* and At*ZIP2*, point to a role of these transporters in root to shoot transport (Milner et al., 2013). The barley transporter HvZIP7 is important for zinc uptake but is localized in root vascular tissue suggesting a similar role in promoting root to shoot transport (Tiong et al., 2013). In rice, OsZIP1 and OsZIP3 have been proposed to be involved in root zinc uptake whereas OsZIP 4, OsZIP5, and OsZIP8 could be involved in root to shoot translocation (Bashir et al., 2013).

After uptake, zinc is in living cells with a neutral pH, a condition in which zinc is prone to bind to a multitude of organic molecules present, which severely restricts its free mobility. Zinc therefore travels between living cells strongly bound to a symplastic metal chelator, which at least in *A. halleri* is the metabolite nicotianamine (Deinlein et al., 2012). The zinc-nicotianamine complex is transportable and can diffuse between cells in the root symplast, which are interconnected by plasmodesmal bridges, toward the xylem, the dead vascular tissue leading to the shoot. The Casparian strip is an impermeable diffusion barrier present in the root apoplast. In the dicot *Arabidopsis* it is present as a layer of lignin (Naseer et al., 2012), which by surrounding the cells of the root endodermis divides the root apoplast in two, an outer apoplast which includes the cell walls of the cortex and extends to the soil solution, and an inner apoplast, which includes the xylem of the central stele. In other plants, such as in monocot cereals, an additional diffusion barrier is present in the exodermis above the cortex and below the endodermis. Cereals thus possess basically two layers of Casparian strips that divide the root into three apoplasts. In the root symplast the transport of zinc is restricted by sequestration for storage into the vacuole, an import requiring active transporters. Two MTPs (*M*etal *T*olerance *P*rotein) AtMTP1 and AtMTP3 have been implicated in this process (Desbrosses-Fonrouge et al., 2005; Arrivault et al., 2006; Kawachi et al., 2009). The HMA AtHMA3 also seems to function in the vacuole to sequester zinc (Morel et al., 2009).

## XYLEM LOADING IN ROOTS

In order to enter the xylem, the dead vascular tissue leading to the shoot, zinc has to be exported from the symplast, which requires active transporters. *A. thaliana* HMA 2 and 4 (AtHMA2 and AtHMA4, respectively), which are primary active zinc pumps, are involved in such loading of the root xylem (Hussain et al., 2004; Verret et al., 2004; Sinclair et al., 2007). AtHMA4 is equipped with an intracellularly exposed zinc-binding domain that may signify post-translational regulation of pump activity in response to a cytoplasmic zinc sensor (Baekgaard et al., 2010). Also, AtHMA2 is known to be regulated at the transcriptional level in response to zinc availability (van de Mortel et al., 2006); both kind of regulation could ensure a tightly controlled xylem loading step. The importance of HMA4 in root-to-shoot translocation of zinc is clearly seen in *A. halleri *where the zinc hyperaccumulation trait strictly depends on AhHMA4 pumping zinc into root xylem vessels (Hanikenne et al., 2008). In rice, the homolog OsHMA2 has also been shown to be involved in root-to-shoot translocation of zinc (Satoh-Nagasawa et al., 2012; Takahashi et al., 2012). In *A. thaliana *other transporters also play a role in xylem loading. At least one member of the Plant Cadmium Resistance family, AtPCR2, is involved in root-to-shoot translocation of zinc (Song et al., 2010) and recently AtZIP1 and AtZIP2 have been implicated in this process (Milner et al., 2013).

Once in the xylem, where pH is slightly acidic (around pH 5.5), zinc can be transported as a free cation. In the zinc hyperaccumulator *Sedum alfredii*, zinc is predominantly transported in the xylem as aqueous zinc. Noteworthy, however, in this plant a significant proportion (around 40%) is associated in the xylem sap with its predominant organic acid, citric acid (Lu et al., 2013b). In *A. halleri*, the zinc mimic cadmium is transported in the free ionic form only (Ueno et al., 2008). In rice, deoxymugineic acid has been associated with long-distance transport of zinc (Suzuki et al., 2008), but it is not known whether the deoxymugineic acid-zinc complex is present in the xylem.

## XYLEM UNLOADING

Zinc is not transported directly to the developing seed by the xylem. Typically, in order to reach the seed, zinc first makes a detour to the leaves. Here it is taken up from the xylem into living xylem parenchyma cells of the leaf symplast. This process is mediated by transport proteins yet to be characterized. Subsequently, zinc is exported from mesophyll cells into the leaf apoplast from where it is loaded into the phloem, which provides the vascular route to the developing seeds. Because of their expression in the vasculature, AtIRT3 (Lin et al., 2009) and OsZIP4 (Ishimaru et al., 2005) may be implied in xylem unloading and/or the phloem loading processes (see below). In grasses, such as rice, a complicated network of vascular bundles in the nodes of the stem allows for a more direct transfer of zinc from the xylem to phloem strands leading to the panicle (Yamaguchi et al., 2012).

## PHLOEM LOADING

The vascular tissue of the phloem is the only route for zinc leading to the developing seeds. Loading of zinc into the phloem typically takes places in leaves. During senescence, zinc is remobilized from withering leaves in order to be allocated to reproductive tissues where demand becomes high. This process requires zinc transport out of mesophyll cells into the leaf apoplast and subsequent transport into the phloem. Before zinc can leave the mesophyll it has to leave the chloroplast, where it plays an important process in photosystem II, and the vacuole, where excess zinc is stored. In *Arabidopsis *HMA1 is localized to the chloroplast envelope and involved in the export of zinc (Kim et al., 2009). This function has also been shown for HvHMA1, where HMA1 has been implied in the remobilization of zinc (Mikkelsen et al., 2012). During zinc remobilization, active transport is not required for zinc in order to leave the vacuole because of the inside positive membrane potential across the tonoplast. In cereals, the remobilization of zinc in the plant during the period of grain loading is not restricted to leaves but also occurs from stems, peduncles, florets and rachis (Walker and Waters, 2011). How zinc is transported out of leaf mesophyll cells is not known.

How zinc enters the phloem is also not known. Transport proteins of the Yellow Stripe-Like (YSL) family, which appear to transport complexes of zinc and nicotianamine across membranes, are highly expressed in senescing leaves (Waters et al., 2006; Curie et al., 2009), and might play a role in transporting zinc into the phloem. Among these, at least AtYSL1 and AtYSL3 are required for allocation of zinc to the developing grain (Chu et al., 2010). Other members of the YSL family might also be involved in the translocation of zinc. AtYSL4 and AtYSL6 have been described as iron transporters localized to organelles, either the chloroplast (Divol et al., 2013) or other endomembrane systems (Conte et al., 2013).

The pH of the phloem is slightly alkaline (Dinant et al., 2010), and therefore zinc has to travel in the phloem bound to a metal chelator, which often appear to be nicotianamine (Curie et al., 2009; Nishiyama et al., 2012).

## PHLOEM UNLOADING IN DEVELOPING SEEDS

The mechanisms involved in phloem unloading and post-phloem movement of zinc in the developing seed are not well understood, but a model has been proposed based on the findings of several apoplastic barriers in the *Arabidopsis* seed. The**seed consists of three genetically distinct tissues: the maternal seed coat, the zygotic embryo and the triploid zygotic endosperm. It is anticipated that such diverse tissues do not contain any symplastic connections and apoplastic barriers are likely to be separating these three tissues in *Arabidopsis* (Stadler et al., 2005). Phloem unloading in the developing seed is believed to be symplastic into a phloem-unloading domain, which has symplastic connections to the entire seed coat (Stadler et al., 2005; Werner et al., 2011). Another apoplastic barrier seems to be present in the seed coat where the outer two cell layers (the outer integument) lacks symplastic connectivity with the three inner cell layers of the seed coat (the inner integument) (Stadler et al., 2005). This gives rise to three apoplastic barriers in the *Arabidopsis* seed where active transport system(s) yet to be identified must be present for translocation of zinc to the embryo: (1) between the outer and inner integuments in the seed coat, (2) between the seed coat and the endosperm, and (3) between the endosperm and the embryo. As active transport is required for zinc in order to escape living cells having a membrane potential highly negative on the inside, zinc efflux from symplasts in the seeds resemble the situation of xylem loading in the root.

The architecture of a cereal grain differs substantially from an *Arabidopsis *seed. In barley nutrients are also unloaded symplastically from the phloem, but they do not end up in the seed coat as in *Arabidopsis*. Instead the nutrients flow to highly specialized nucellar projection transfer cells. In the developing barley grain, apparently a single apoplastic barrier is present between the maternal transfer cells and the filial endosperm (Borg et al., 2009). In the wheat grain, an additional apoplastic barrier for Zn has been identified between the stem tissue rachis and the developing grain, which most likely is linked to unloading from the xylem of the stem and subsequent loading of the phloem leading to the grain (Wang et al., 2011). Whether this is a general feature of cereal grains remains to be shown. In a microarray study from laser capture microdissection of the developing barley grain it was found that *HvHMA2* was almost exclusively expressed in the transfer cells in the developing barley grain (Tauris et al., 2009). The expression of *HvHMA2 *in the transfer cells makes HvHMA2 a prime candidate for export of zinc from the mother plant into the endosperm cavity (Tauris et al., 2009). The zinc sensitivity of the *Saccharomyces cerevisiae zrc cot1 *mutant is**alleviated following heterologous expression the heterologous expression of *HvHMA2, *supporting the notion that HvHMA2 functions as a zinc export pump (Mills et al., 2012). Transporters of the MTP family might also be involved in controlling the amount of zinc crossing the apoplastic barriers in the developing seed by sequestering zinc into the vacuole on either side. Several MTPs are expressed in barley (Tauris et al., 2009) and *Arabidopsis *(Desbrosses-Fonrouge et al., 2005) seeds.

## NICOTIANAMINE – THE INTERCELLULAR AND PHLOEM MOBILE ZINC-LIGAND

Apart from the transport across apoplastic barriers in the root and seed requiring membrane transporters, the intercellular and long-distance transport of zinc influences the loading of zinc to the seed. Due to its low solubility at neutral and alkaline pH, zinc is not present as free ions, but will be bound to a ligand. Nicotianamine, in particular, has emerged as a major zinc chelator in plants although it also chelates other metals such as iron and copper (Clemens et al., 2013).

In the zinc hyperaccumulator *A. halleri*, nicotianamine synthase (NAS) is highly expressed and nicotianamine has been shown to be involved in root-to-shoot translocation of zinc with a five-fold decrease in *NAS2*-RNAi lines. This has been ascribed to a role for nicotianamine in the symplastic mobility of zinc in the root toward the pericycle cells (Deinlein et al., 2012). The phenotypes of the *A. halleri NAS2*-RNAi lines (Deinlein et al., 2012) and the *HMA4*-RNAi lines (Hanikenne et al., 2008) are somewhat similar. Both show reduced root-to-shoot translocation and accumulation of zinc in the root pericycle cells due to reduced xylem loading. This makes it very interesting to look further into the connection between nicotianamine and HMA4.

Nicotianamine seems to be highly important for the intercellular mobility of zinc but might also be involved in enhancing the mobility of zinc in the phloem essential for getting zinc to the developing seed. Several reports have shown that the constitutive overexpression of *NAS* genes in rice is able to increase the zinc concentration in the rice grain by in average twofold, also in the polished grain (Lee et al., 2009; Masuda et al., 2009; Johnson et al., 2011; Lee et al., 2011). This probably happens because the enhanced level of nicotianamine increases the phloem mobility of zinc, thus enhancing the flow of zinc to the nucellar projection in the rice grain. Likewise, overexpression of *NAS *in combination with ferritin affects Fe accumulation in the rice endosperm (Wirth et al., 2009). Nicotianamine has been shown to be the major zinc-ligand in the leaf phloem sap of wild-type rice (Nishiyama et al., 2012) and one of the main limitations in getting zinc to the developing seed has been identified as being its transport in the phloem (White and Broadley, 2011). Also, it was found that zinc bound to nicotianamine in the endosperm is highly bioavailable (Lee et al., 2011). A significant problem concerning zinc biofortification is that transporters for zinc often co-transport the zinc mimic cadmium. However, nicotianamine seems to be highly specific for zinc over cadmium and, accordingly, rice plants overexpressing *OsNAS* do not show an increase in cadmium (Lee et al., 2009, 2011).

## CONCLUSION

Taken together, the transport of zinc from the root to the shoot and further to the developing seed results from an interplay not only between different membrane transporters but also between the mobility of zinc both intercellularly and between organs by binding to ligands. The presence of multigenic factors that control the amount of zinc ending up in the edible parts of the seed makes it a challenging future to increase bioavailable zinc in the edible parts of cereal grains. The substantial increase in the grain zinc concentration found in plants overexpressing *NAS *genes makes nicotianamine an interesting target for zinc biofortification.

## Conflict of Interest Statement

The authors declare that the research was conducted in the absence of any commercial or financial relationships that could be construed as a potential conflict of interest.
